# *COI* Sequencing from Guano Improves Bat Diversity Research: The First Report from Bosnia and Herzegovina

**DOI:** 10.3390/biology15151313

**Published:** 2026-08-06

**Authors:** Šejla Goletić Imamović, Teufik Goletić, Ilma Terzić, Naida Kapo, Vojo Milanović, Adis Softić, Zinka Maksimović, Asja Bubić, Dunja Rukavina

**Affiliations:** 1 Veterinary Faculty, University of Sarajevo, 71000 Sarajevo, Bosnia and Herzegovina; sejla.goletic@vfs.unsa.ba (Š.G.I.); ilma.terzic@vfs.unsa.ba (I.T.); naida.kapo@vfs.unsa.ba (N.K.); adis.softic@vfs.unsa.ba (A.S.); zinka.maksimovic@vfs.unsa.ba (Z.M.); asja.bubic@vfs.unsa.ba (A.B.); dunja.rukavina@vfs.unsa.ba (D.R.); 2 Department of Biology, Faculty of Science, University of Sarajevo, 71000 Sarajevo, Bosnia and Herzegovina; milanovic.vojo9@gmail.com

**Keywords:** *COI*, sequencing, bats, guano, Bosnia and Herzegovina

## Abstract

Bats are one of the most widespread, but also the least researched groups of mammals. Their guano is an important source of DNA because it is often readily available even if bats are not, and it may be used to reliably identify different species of bats. According to the most recent data, there are 31 species of bats recorded in Bosnia and Herzegovina, identified by the morphometric and acoustic methods. This study had the goal of studying bat diversity in Bosnia and Herzegovina by sequencing the *COI* gene from guano samples and comparing the results with the results of morphometric measurements. A total of 10 bat species were identified through morphometric analysis, while 13 bat species were identified through the sequencing of *COI* gene from guano samples, including a new species for the country: *Rhinolophus mehelyi*. Sequencing has increased the number of species recorded on each locality and resolved inconclusive morphological findings except in the case of *M. myotis/blythii*. The results indicate that the biodiversity of bats in Bosnia and Herzegovina is larger than estimated and that *COI* sequencing from guano can be used for regular monitoring and for identification of sites of special importance to bats.

## Simple Summary

The biodiversity of bats in Bosnia and Herzegovina has only been assessed by methods that require capture and/or the analysis of echolocation signals, thus giving and incomplete picture of the true biodiversity. To address this, we aimed to measure bat diversity by analyzing DNA from bat droppings (guano) and comparing the results of this advanced genetic method to traditional morphological measurements of captured bats. The results revealed that while the morphological method identified only 10 bat species, the DNA analysis successfully identified 13 species—including Mehely’s horseshoe bat, a species never recorded in the country before. Furthermore, DNA testing clarified uncertain morphological identifications and discovered more species at every location. The study concludes that Bosnia and Herzegovina hosts a much richer diversity of bats than previously thought, and proves that non-invasive DNA sampling from guano is a highly effective tool for routine bat monitoring and provides an easy, harmless way for conservationists to pinpoint and protect critical natural habitats, ensuring the survival of these ecologically vital animals and maintaining healthy local ecosystems.

## 1. Introduction

Bats (order Chiroptera) represent one of the most species-rich orders of mammals, comprising 1511 currently recognized species adapted to a wide range of environments [1]. These flying, relatively small mammals are active at night, and among numerous species there are very small morphological differences, which can make research efforts difficult, especially in the context of identification and conservation of the rarest and most endangered species. Bat species identifications are often based on morphometry and acoustic analysis of echolocation. However, both methods have significant limitations that complicate the problem of identifying bat species [2].

Acoustic identification of bat species is based on the recording of echolocation signals that can differ in frequency and shape between different species [3]. Recording and analyzing echolocation signals has proven important in conservation efforts because this is a non-invasive method that can provide insight into the presence of certain bat species at a given location. However, a fundamental limitation of this method is that the echolocation signals of many species are highly variable and often overlap [4]. Therefore, proper species identification using acoustic methods requires a high level of knowledge and experience, but even in this case the results are not always objective and can be difficult to confirm, even if they are recorded in the form of a sonogram that can be subsequently analyzed [5].

Morphometric identification of bat species implies identification of the species based on the measurements of certain parts of the body, including the length of the forearm, the third and fifth toes, length and width of the ear and tragus, length and shape of the upper row of teeth, etc. [5]. Regardless, it is not always possible to identify the bat species solely on the basis of morphology. Some species, such as those of the whiskered bats group, are so similar to each other that it is difficult to distinguish between them, and in certain cases individuals differ from the usual appearance of the species to such an extent that it is not possible to make a precise identification [6]. High levels of intraspecific variability have been reported in some species, and identification keys generally cannot capture all possible variation [7]. Additionally, this method requires catching bats, which involves the use of mist nets or taking bats from their hibernating colonies. In the first case, it is only possible to identify caught bats, while some species that escape the nets remain unrecorded, thus giving an incorrect picture of species abundance in a locality [8]. In the second case, bats are much easier to catch because they are much slower, but this implies disturbing the colonies, which can adversely affect the survival of all bats present [9]. Therefore, although the morphometric method can be considered more accurate than the acoustic method, its major drawback is that it involves disturbing bats, which can endanger their well-being.

Numerous challenges related to the identification of bat species by acoustic and morphometric methods have been overcome by using molecular methods of identification. DNA barcoding involves the sequencing of genetic markers such as mitochondrial genes, which is an approach used in the identification of numerous mammalian species [10,11]. By using DNA barcoding the number of known species in the western Palearctic region (Europe and North Africa) has increased from 37 to 51 [12]. The samples most often used for this purpose are small pieces of flight membrane (wing punches) with a diameter of 2 mm, which subsequently heal [6]. However, this type of sampling is invasive and requires the capture of bats, which is stressful for the animals, and provides an incomplete picture of the presence of bats in an area because uncaptured species remain unrecorded. Species identification using DNA isolated from feces has proven to be an adequate alternative to morphometric methods, but also to sampling pieces of flight membrane. For bat identification, guano has proven to be an adequate and, from an animal welfare perspective, particularly important, non-invasive sample, from which it is possible to obtain reliable information about the bat species present in a given locality [13]. PCR-based sequencing methods have enabled the identification of bat species at the molecular level using markers such as the NADH dehydrogenase subunit I (*nd I*) gene [12] and the cytochrome c oxidase subunit I (*COI*) gene [2,14]. In addition, *COI* is a standardized marker for the identification of different animal species, and as such is the most widely used in online databases. In addition, low intraspecific and high interspecific variation within the *COI* marker has been reported in bats, and it has been successfully used for taxonomic species identification and discovery of new bat species [2,15]. In addition to the fact that data obtained through sequencing are more reliable than morphological measurements, it is possible to obtain data from guano on the biodiversity and diet of the species present without the need to capture, measure, or even observe the animals that produce it [16,17].

According to the data listed in the Red List of Fauna of the Federation of Bosnia and Herzegovina (bos.: Crvena lista Federacije Bosne i Hercegovine) and Red List of protected species of flora and fauna of the Republic of Srpska (bos.: Crvena Lista zaštićenih vrsta flore i faune Republike Srpske) there are a total of 27 species of bats in Bosnia and Herzegovina (B&H), classified in the families Vespertilionidae, Rhinolophidae and Molossidae. All species are considered either as vulnerable or endangered [18,19]. However, recent surveys have increased the number of recorded bat species in the country to 33 [20]. Despite this increase, the distribution and conservation status of several species remain insufficiently understood due to limited research. Until now, bat monitoring in B&H has relied predominantly on acoustic surveys and morphometric identification. Having in mind the limitations and drawbacks of this method, this study had the goal of identifying bat species by sequencing the *COI* gene from guano samples and comparing these results with the results of morphometric measurements in order to estimate B&H bat biodiversity more precisely.

## 2. Materials and Methods

### 2.1. Sample Collection

The collection of guano samples from live bats, as well as the collection of guano from sites inhabited by bats, was approved by the Ethics Committee of the Faculty of Veterinary Medicine, University of Sarajevo (Decision No. 07-03-262-2/22 of 7 April 2022). No bats suffered injuries and all were released alive after sampling. If caught, mothers with pups were released immediately without sampling.

**Figure 1:**
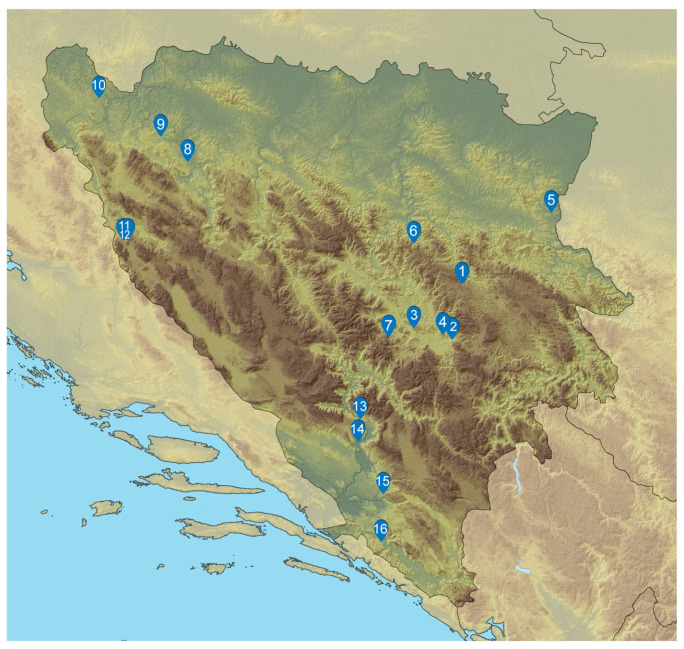
Sampling localities included in this study. 1: Protected landscape Bijambare (Middle cave), 2: Trebević (tunnels), 3: Vela (Rakovica), 4: Sarajevo, 5: Dardagani quarry (Zvornik), 6: Nature monument Tajan—a cave in Duboka Tajašnica, 7: Ober pit (Kreševo), 8: Hrustovača cave (Sanski Most), 9: Fajtovačka cave (Fajtovci), 10: Cave Ada (Jezersko), 11: Vodena cave (Resanovci, Bosansko Grahovo), 12: Ledenica cave (Resanovci, Bosansko Grahovo), 13: Salakovac (tunnels), 14: Mostar, 15: Stolac, 16: Gradina tunnel.

Sampling was carried out in the three-year period, during each spring and autumn, when the bat activity is increased and guano has accumulated, at a total of 16 sites (Figure 1). The sites were selected based on existing data, i.e., estimates, on the size of bat colonies and their biodiversity, but also on their proximity to human habitats and the rate of visitation by humans and their animals (e.g., tourist caves). The additional information about all sampling sites (coordinates and type of habitat) is available in Supplementary Table S3. At each site, samples were collected at least twice in different periods of the year, and the geographical coordinates and altitude were recorded. No bats were captured at the Mostar and Gradina tunnel sites, and only guano samples were collected.

The study included 474 bat guano samples from the floor of their habitats, as well as 148 fresh guano samples (2–3 pellets) and 190 anal swabs from 190 captured bats. A detailed list containing the number of samples collected at each locality is available in Supplementary Table S4.

The capture of individuals was carried out by chiropterologists with previous experience in working with bats. The captured individuals were placed in clean cotton bags for half an hour, after which the produced guano and anal swabs were collected from them, and then they were released unharmed at the capture site. Anal swabs, as well as the guano produced by the captured bats, were collected using OmniSwab swabs (Qiagen, Hilden, Germany) which were immediately placed in sterile 2 mL Eppendorf DNA LoBind tubes (Eppendorf, Hamburg, Germany) after sampling.

In addition to anal swabs and the freshly produced guano, guano present on the floor was also collected at each site visited. Each individual sample did not contain more than 2 g of guano, which was done according to the criteria of the nucleic acid extraction kit used. Floor guano samples were separated based on their location within the sites themselves (piles directly under a specific bat colony, closer or further from the entrance to the site, etc.). Immediately after sampling, guano was placed in sterile 2 mL Eppendorf DNA LoBind tubes (Eppendorf, Hamburg, Germany) and, together with the swabs, transported to the laboratory of the University of Sarajevo–Veterinary Faculty in a portable refrigerator with ice. All samples were stored at −20 °C until further processing.

### 2.2. Morphological Identification of Captured Bats

Live bats were captured with hand nets and mist nets (Ecotone, Gdynia, Poland). Morphological identification of bats was performed using an identification key [21]. (Each captured individual was, if possible, identified to the species level based on measured morphological features including forearm length (FA), third finger length (D3) and fifth finger length (D5). Morphological measurements were taken from a total of 190 captured bats using a Digimatic digital caliper (Mitutoyo, Kawasaki, Japan). The measurements of the individuals, as well as the capture, were carried out by experienced chiropterologists. The sex and age (juvenile or adult) of each caught individual were determined and recorded following a method previously described elsewhere [5,21].

### 2.3. Nucleic Acid Extraction, Quantification and COI Sequencing

Total nucleic acid extraction from all individual guano samples was performed using the Fast DNA Stool Mini kit (Qiagen, Hilden, Germany) according to the manufacturer’s instructions. Additionally, total nucleic acid extraction from all collected anal swabs was performed using the QIAamp DNA Mini kit (Qiagen, Hilden, Germany). Before the extraction with the kit, the tip of each swab was cut with sterile scissors and inserted into a sterile 1.5 mL Eppendorf tube (Eppendorf, Hamburg, Germany) with 0.8 mL of sterile PBS. The tubes were then vortexed briefly on an IKA Matrix Orbital thermoshaker (IKA Werke GmbH & Co. KG, Staufen im Breisgau, Germany) and left for 10 min at room temperature to allow the precipitate to settle, after which 140 µL of the supernatant was removed and used for further extraction process according to the manufacturer’s instructions.

After extraction, the concentration of total nucleic acids in each nucleic acid extract was measured using a fluorimetric Qubit 4 device and a Qubit dsDNA HS (High Sensitivity) Assay kit (Invitrogen, Waltham, MA, USA), without changing the protocol formulated by the manufacturer. Each sample was tested in duplicate, and those samples for which ≥1 μg of total nucleic acids were measured were used for further analysis. Extractions from anal swabs and fresh guano from the same bat were pooled and treated as a single sample in the downstream analyses. A total of 60 pooled anal swabs/fresh guano and 120 guano samples from the site floor were used in the molecular analyses, covering all sites in all sampling periods. Additional criteria for sample selection were the previously recorded bat diversity at the sampling site and the number of samples collected at each locality. Detailed information about how many samples per locality were analyzed is given at Supplementary Table S4.

For the purpose of bat species identification, 202 bp long fragments of the mitochondrial *COI* gene were amplified from DNA samples by PCR [2]. Before the reaction, all samples with a concentration of >1 μg were diluted to a concentration of 1 μg to reduce the likelihood of inhibition of the PCR reaction. The protocol according to Walker et al. [2] was modified as follows: the PCR reaction consisted of 12.5 μL Q5 High-Fidelity 2X Master Mix (New England Biolabs, Ipswich, MA, USA), 6 μL nuclease-free water, 4 μL of the sample, and 1.25 μL each of 10 μM primers SFF_145f (5′-GTHACHGCYCAYGCHTTYGTAATAAT-3′) and SFF_351r (5′-CTCCWGCRT-GDGCWAGRTTTCC-3′) (Eurofins Scientific, Luxembourg City, Luxembourg). The thermal profile consisted of an initial denaturation at 98 °C for one minute, followed by 35 cycles of denaturation at 98 °C for 10 s, annealing at 60 °C for 30 s, and extension at 72 °C for 30 s, followed by a final extension at 72 °C for 2 min. Amplification of the *COI* gene fragments for sequencing was performed in a T100 Thermal Cycler (Bio-Rad, Hercules, CA, USA). The PCR products were then prepared for sequencing on a MinION Mk1C device according to the manufacturer’s instructions: The Native Barcoding Kit 1–12 (EXP-NBD104), Native Barcoding Kit 13–24 (EXP-NBD114), and Ligation Sequencing Kit (SQK-LSK109) (Oxford Nanopore Technologies—ONT, Oxford, UK) were used to prepare the DNA library, which was loaded onto a R9.4.1 flow cell (ONT, Oxford, UK) using the Flow Cell Priming Kit (ONT, Oxford, UK). The sequencing run lasted 12 h, during which the high-accuracy basecalling of FAST5 files and demultiplexing was performed in real time in MinKNOW v.20.10.3 (Guppy v.4.2.2), with a Q score set to 9, and selected options “trim barcodes” and “barcode both ends”. The minimum barcoding score was set to 70.

### 2.4. Bioinformatic Analysis

After the completion of a sequencing run, quality control was performed with the FastQ Control Experiment workflow module in EPI2ME v.3.7.3. software (MinKNOW Core v.4.1.2), after which only those reads with a Q score ≥ 12 were used in downstream analysis.

PCR amplification of the *COI* fragment yielded amplicons of 202 bp, and after quality control, too short (<170 bp) and too long (>230 bp) reads were removed using the seqkit (v2.8.0) tool in WSL Ubuntu 22.04.5 [22]. In addition, a total of 36 reference *COI* sequences (accession numbers listed in Supplementary Table S1) were downloaded from the online database BOLD Systems (https://boldsystems.org/, accessed on 24 October 2025), including sequences of all bat species recorded so far in B&H (31) and sequences of species that may be present (5) based on available data from neighboring countries and the rest of Europe. The purified *COI* fragments were mapped to all 36 *COI* sequences using the Minimap2 (v2.24-r1122) tool [23] in WSL Ubuntu 22.04.5, and the resulting Sequence Alignment Map (SAM) files were visualized in the program Geneious Prime (available at http://www.geneious.com/, v.2025.0.3). Preliminary species identification was performed based on the coverage of mapped fragments to the reference *COI* sequence at positions 151–352 bp, which corresponds to the part of the *COI* fragment that was amplified during the PCR reaction. Consensus sequences in FASTA format were extracted from SAM files using the extract consensus option of the Geneious Prime v.2025.0.3 program. Species identification was performed by inserting the obtained consensus sequences into the NCBI Basic Local Alignment Search Tool (BLASTn, https://blast.ncbi.nlm.nih.gov/Blast.cgi, accessed on 27 October 2025) and assessing the level of similarity with *COI* sequences of different bat species stored in this database. In addition, confirmation of the results obtained by BLAST analysis was performed by inserting the consensus sequences into the BOLD Identification Engine (https://v3.boldsystems.org/index.php/IDS_OpenIdEngine, accessed on 27 October 2025) and searching its Species Level Barcode Records database based on sequence similarity. Species identification was considered reliable if a search of both BLAST and BOLD databases yielded a consensus sequence match of >99% with sequences of identical species stored within these databases. All obtained *COI* consensus sequences were aligned with reference *COI* sequences of identified species using the ClustalW module in MEGA11 11.0.13 software [24]. In the case of the presence of two identical sequences from the same locality, only one sequence was retained. All obtained *COI* sequences with supporting data (species, sampling date) were uploaded to the BOLD Systems database and are available at the following link: https://portal.boldsystems.org/recordset/DS-BATBIH25, accessed on 27 October 2025.

The phylogenetic tree was constructed in MEGA11 11.0.13 software [24] using the Neighbor-Joining (NJ) method, while evolutionary/genetic distances were calculated using the Tamura 3-parameter [25] nucleotide substitution model with 1000 bootstrap tests. The selection of nucleotide substitution models was performed using the programs jModelTest 2.1.10 [26,27] and IQTREE [28,29]. The *COI* sequence for the species *Pteropus hypomelanus* (small flying fox, family Pteropodidae) was downloaded from the BOLD database (ID: ABBSI330-11) and used as an outgroup when constructing the phylogenetic tree.

In order to determine the genetic distances within the species and among different species, intraspecific and interspecific genetic distances were calculated using MEGA11 11.0.13 software [24]. The Tamura 3-parameter model with 1000 bootstrap tests was used, assuming that both transitions and transversions occur uniformly at all nucleotide positions in the sequences [25].

## 3. Results

### 3.1. Morphological Identification of Bat Species

The species, sex and number of caught, identified and sampled individual bats at a given sampling locality are listed in Table 1. Data is given for 14 locations where bats were caught and sampled; the localities Mostar and Gradina tunnel were not included in the analysis since no bats were caught there. All caught bats were sexually mature adults, and a total of 105 males and 85 females were recorded. A total of ten bat species was recorded, eight of which were successfully identified: *Barbastella barbastellus*, *Miniopterus schreibersii*, *Myotis capaccinii*, *Myotis myotis*, *Rhinolophus euryale*, *Rhinolophus hipposideros*, *Rhinolophus ferrumequinum* and *Tadarida teniotis*. Additionally, one recorded individual was identified to the genus level as *Myotis* sp. Two individuals were designated as *Myotis mystacinus/alcathoe* because they could not be reliably identified as either *M. mystacinus* or *M. alcathoe* due to the significant overlap in morphological features and ecological niches that these species inhabit. Similarly, one individual from the locality Dardagani quarry was designated as *M. myotis/blythii* due to the ambiguity of its morphological features.

The most abundant species, as determined by morphological measures and markers, is *R. hipposideros*, present at 12/14 (85.71%) sites, while the species *B. barbastellus*, *M. mystacinus/alcathoe*, *M. myotis*, *M. myotis/blythii*, *R. euryale* and *T. teniotis* were present at only one site each (7.14%). Dardagani quarry is the richest sampling site in terms of biodiversity, as seven different bat species were recorded there, which accounts for 70% of the species recorded using morphological methods in this work. In addition, three of the seven species—*M. myotis*, *M. myotis/blythii* and *R. euryale*, were not recorded at other sites.

### 3.2. Molecular Identification of Bat Species

Sequencing of the *COI* fragment generated an average of 97,542 reads per sample, with an average length of 260 bp and an average coverage of 1085 among all samples. On average, 53% of the reads were retained from each sample and used for sequence mapping and assembly.

**Figure 2:**
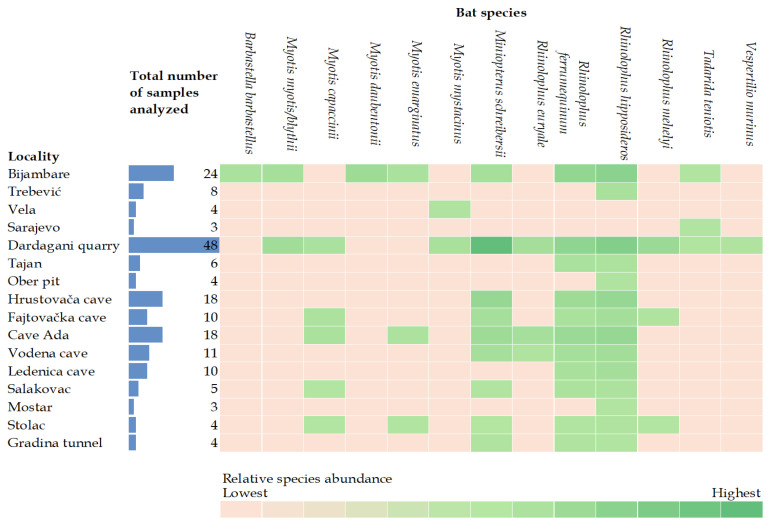
Heatmap of relative abundance of bat species as determined by the sequencing of the *COI* gene fragment from bat guano.

**Figure 3:**
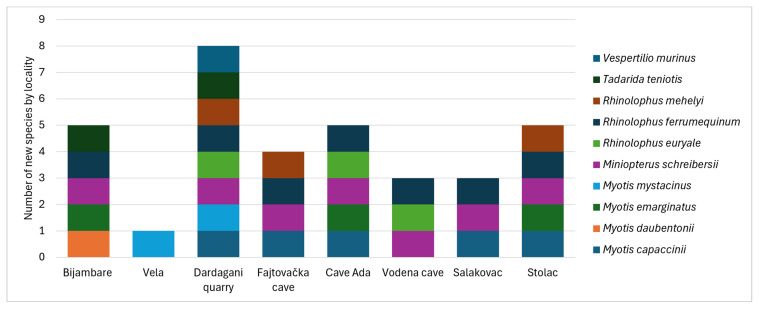
Bat species and the localities where they were recorded for the first time.

Analysis of *COI* fragments of bat sequences from guano collected from 16 localities identified a total of 13 species: *Barbastella barbastellus*, *Myotis myotis/blythii*, *Myotis capaccinii*, *Myotis daubentonii*, *Myotis emarginatus*, *Myotis mystacinus*, *Miniopterus schreibersii*, *Rhinolophus euryale*, *Rhinolophus ferrumequinum*, *Rhinolophus hipposideros*, *Rhinolophus mehelyi*, *Tadarida teniotis* and *Vespertilio murinus* (Figure 2). Bats that were identified as *M. mystacinus/alcathoe* by morphometric analysis were identified by *COI* analysis as *M. mystacinus*, and *Myotis* sp. was identified as the species *Myotis emarginatus*. By comparing the sequences of the *COI* fragment of the species *M. blythii* and *M. myotis*, it was determined that these two species cannot be distinguished using *COI* markers, and they are presented as a joint finding (*Myotis myotis/blythii*). In addition, Figure 3 displays the bat species recorded for the first time at certain locations, i.e., species that prior have not been recorded by morphometric or acoustic methods at these locations. New species were recorded at a total of eight (50%) locations. A particularly important finding is the species *Rhinolophus mehelyi* (Mehely’s horseshoe bat), which has not been recorded on the territory of B&H to date. It was recorded at the Dardagani quarry, Fajtovačka cave and Stolac.

By implementing *COI* sequencing, the recorded species diversity was increased for every locality, with sequencing from guano enabling detection of at least one additional species at 11/16 (68.75%) localities. An increase ≥50% in species richness was recorded at eight localities. The detailed overview is species richness per locality is presented in Table 2.

**Figure 4:**
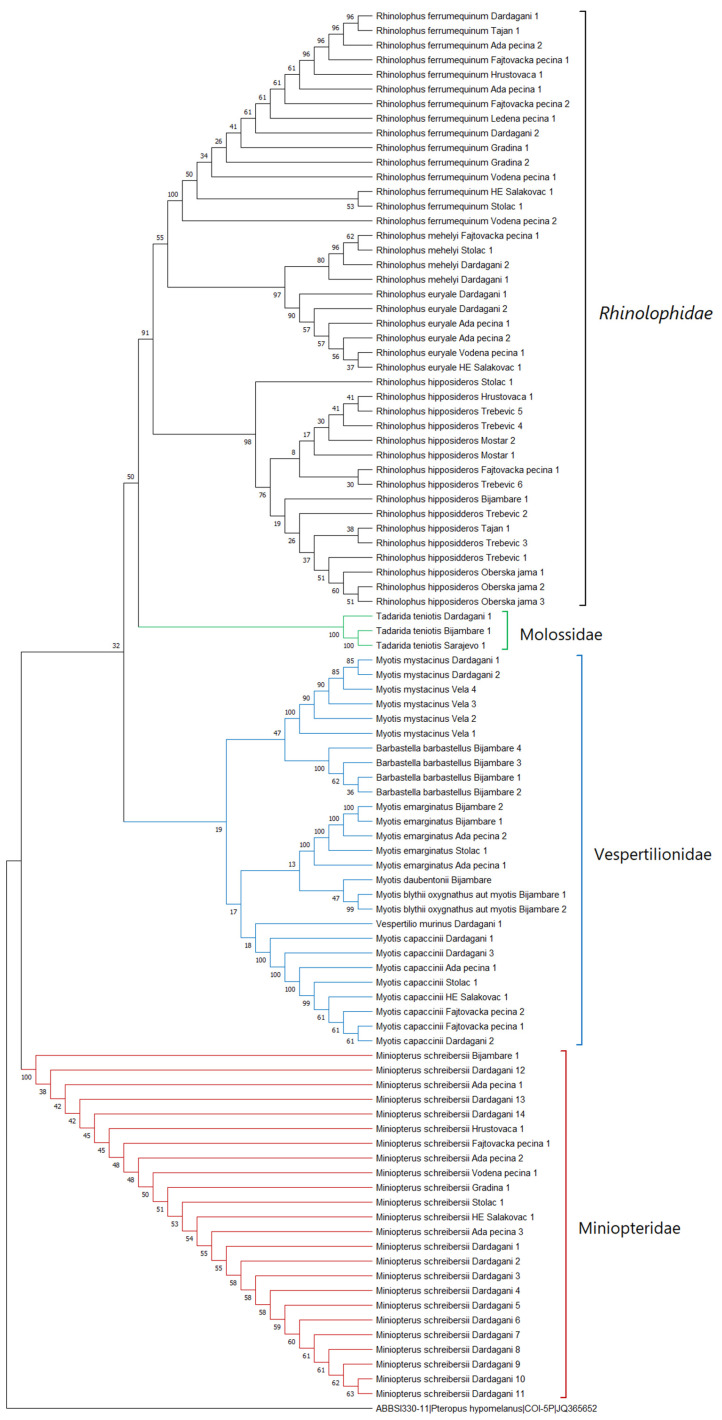
Phylogenetic tree of bat *COI* sequences from guano collected at various sites in B&H. The four families are marked with brackets and branches in matching colors: Rhinolophidae—black, Molossidae—green, Vespertilionidae—blue and Miniopteridae—red. The phylogenetic tree was constructed in MEGA11 11.0.13 software using the Neighbor-Joining (NJ) method, while evolutionary/genetic distances were calculated using the Tamura 3-parameter nucleotide substitution model with 1000 bootstrap tests. The bootstrap values are displayed at the nodes. The *COI* sequence for the species *Pteropus hypomelanus* (small flying fox, family Pteropodidae) was downloaded from the BOLD database (ID: ABBSI330-11) and used as an outgroup.

The most abundant species is *Rhinolophus hipposideros*, whose presence was determined at 87.5% (14/16) of the sampling sites. The least abundant species are *Barbastella barbastellus* and *Myotis myotis/blythii,* which were recorded only at the Protected landscape Bijambare, and the species *Vespertilio murinus*, recorded only at the Dardagani quarry. The Protected landscape Bijambare and the Dardagani quarry are characterized by the highest diversity of bat species, as eight and ten species were recorded at both sites, respectively, which makes up 61.5% and 76.9% of the total bat species recorded in this study. In terms of biodiversity, the sites Trebević, Vela, Sarajevo, Ober pit and Mostar displayed the poorest biodiversity, with one bat species recorded at each site. The largest number of new species per locality, five of them, were recorded at the Stolac locality. The most abundant bat family is the Vespertilionidae family with seven different bat species recorded, while the families Miniopteridae and Molossidae were represented by one species each. The phylogenetic tree of *COI* sequences of bats in B&H is shown in Figure 4.

The results of the analysis of intraspecific and interspecific genetic distances of different bat species in B&H based on their *COI* sequences are presented in Table 3 and Table S2, respectively. It was not possible to calculate intraspecific genetic distances for the species *Myotis daubentonii* and *Vespertilio murinus*, as both were represented by only one sequence. The lowest intraspecific genetic distance (*d* = 0) within the tested *COI* fragment was recorded for the species *Tadarida teniotis*, *Myotis emarginatus* and *Miniopterus schreibersii*, while the highest intraspecific genetic distance (*d* = 0.0229) was recorded for the species *Rhinolophus hipposideros* (Table 3).

The lowest recorded interspecific genetic distance (*d* = 0.0427) within the tested *COI* fragment is between the species *Rhinolophus mehelyi* and *Rhinolophus euryale*, and the highest recorded interspecific genetic distance (*d* = 0.3050) is between the species *Rhinolophus ferrumequinum* and *Barbastella barbastellus*. The standard deviation is marked in blue and is presented below the diagonal, while the *d* value is marked in black and is located above the diagonal (Table S2).

## 4. Discussion

A total of 10 different bat species were recorded at a sum of 14 localities using the morphometric method of species identification. The species recorded by this method in this work have also been previously recorded at the given sampling localities [30] with the exception of the locality Vela for which no previous information existed. Several individuals could not be identified to the species level: an individual *Myotis* sp. from the locality Bijambare, two *Myotis mystacinus/alcathoe* from the locality Vela and *Myotis myotis/blythii* from the Dardagani quarry. The listed species have very similar morphological features: *M. mystacinus* and *M. alcathoe* belong to the group of so-called whiskered bats, which includes several species with high intraspecific variation, as well as age-dependent variation in fur color [6]. Discrimination between these species is based on the shape of the dentition, the shape of the nostrils, and the length of the snout when compared to the fur [5,21]. Furthermore, the species *M. mystacinus* is so variable that it likely includes several cryptic species [5]. Morphological features of the species *M. myotis* and *M. blythii* overlap to the extent that individuals are often very difficult to distinguish from each other to the researcher inexperienced with morphometric identification, and the existence of hybrids with a mixture of morphological features of both species further complicates identification [31]. The correct identification of a bat species largely depends on the correct measurement and interpretation of certain qualitative features (e.g., fur color, shape of the tragus), and therefore the biggest drawback of this method is that it is subject to the expertise and assessment of the examiner, i.e., human error [7]. Additionally, morphometric identification is stressful for the animal, which can lead to unwanted consequences such as death of the animal or injury caused by the animal trying to free itself. An additional limitation from a biodiversity research perspective is that the number of different bat species recorded at a given site is highly dependent on sampling, which is opportunistic in nature, meaning that species that fly beyond the range of trapping nets remain unrecorded [8]. Although the morphometric method of identification is still the standard for the identification of bat species in B&H and the region, it has numerous shortcomings and as such should not be used alone, but rather in combination with other methods.

The results of this study demonstrated that collecting guano samples for species identification by molecular methods is a very effective monitoring approach. More species were recorded with molecular methods than by visual assessments and/or morphometric analyses, which is confirmed by the results of other studies [2,32,33,34]. If environmental conditions are stable, it is possible to amplify the *COI* segment from guano, regardless of its age. Walker et al. [2] found that the greatest influence on the stability of DNA in guano sampled from caves is relative humidity of the cave, and that in dry caves (about 51% humidity) DNA in guano is stable for over 30 months, while in wet caves (almost 100% relative humidity) DNA is largely degraded after only six months. Caves in B&H are mostly of karst origin with high relative humidity; e.g., in The Middle cave in Bijambare the relative humidity varies between 80 and 95% [35]. However, *COI* fragments were successfully amplified and sequenced from all tested samples, which could be attributed to the fact that guano from all sites was collected twice a year and, therefore, was probably not older than six months. An additional point to consider when analyzing guano samples is the choice of the extraction kit; while Fast DNA Stool Mini Kit successfully extracted the DNA from guano samples, it failed to extract DNA from the anal swabs. For these samples, QIAamp DNA Mini Kit proved to be an adequate substitute kit for DNA extraction.

Sequencing *COI* markers from guano samples at 16 sites determined the presence of 13 bat species, with it being important to note that at eight (50%) sites certain species were recorded for the first time (Figure 3). Among these findings, the most notable one is the finding of the species *Rhinolophus mehelyi*, whose presence was recorded for the first time in B&H, at three different sampling sites. Several factors lend legitimacy to this finding: the habitat of the species *R. mehelyi* includes open spaces such as meadows, pastures and agricultural lands, but also forests [5]. It often forms mixed colonies with other species of the genus *Rhinolophus*, almost exclusively in caves and abandoned mines, which coincides with the sites in B&H where its presence was recorded. *COI* sequences identified as *R. mehelyi* were recorded multiple times on each of the three different sites and in different sampling periods. The quality of the *COI* sequences was high with very good genome coverage (x875). Next, the primers used in this work were tested by Walker and colleagues in a controlled experiment that determined that they can be used to detect rare or poorly represented species in guano. The primers successfully amplified *COI* fragments of rare species whose DNA was present in the samples at dilutions of up to 1:192 [2]. Furthermore, the low intraspecific distance (0.0178 (Table 3) and relatively high (0.0427–0.2847) interspecific distance (Table S2) with other species clearly distinguish it as a separate species. This is also confirmed by the fact that all *R. mehelyi* sequences on the phylogenetic tree (Figure 4) cluster together and form a branch separate from other species. *R. mehelyi* sequences form a smaller cluster with *R. euryale* sequences, with which they have the lowest recorded interspecific distance (0.0427) and with which they often form colonies [5]. Furthermore, analysis of *COI* sequences in the BOLD and GenBank databases gives >99.8% similarity to other *COI* sequences originating from *R. mehelyi*. All of these factors combined indicate that the molecular finding of *R. mehelyi* is reliable. In future monitoring of sites where *R. mehelyi* has been recorded, it is necessary to capture an individual of this species, collect additional samples and analyze additional loci to further confirm the findings from guano and rule out a rare introgression event.

One of the main advantages of species identification by *COI* fragment sequencing is the reliable identification of the species regardless of morphological characteristics, which, in two cases, enabled species identification where morphometric identification was not possible: an individual from the Bijambare locality identified by morphometric analysis as *Myotis* sp. was identified by sequencing and analysis of the *COI* fragment as *Myotis emarginatus*, while two individuals, morphometrically identified as *Myotis mystacinus/alcathoe* were identified by *COI* fragment analysis as *M. mystacinus*. An exception is the finding of *M. myotis/blythii*, where the species could not be reliably identified by either morphometric or molecular methods used. The sequenced *COI* segment corresponding to the species *M. blythii* and *M. myotis* is identical, and this finding was confirmed by comparing the sequences of the corresponding *COI* fragment stored in the BOLD database. For the same reason, the morphological finding of *M. myotis* from Dardagani quarry could not be reliably identified as *M. myotis* or *M. blythii* by the molecular method. Identical *COI* sequences were also noted by Ruedi et al. [15] when identifying the so-called “sister” species *M. myotis/M. blythii*, *M. nattereri/M. crypticus* and *Eptesicus serotinus/E. nilsoni*. A high level of mitogenome similarity was observed in these pairs of closely related species, which is most likely a consequence of ancient, massive mitochondrial introgression, a process in which mitochondrial genes from one species are permanently incorporated into the genome of another species, to the point that complete replacement of mitochondrial DNA (mtDNA) can occur [15,36]. This phenomenon has been previously observed in European *M. myotis* and *M. blythii*, whose mitochondrial haplotypes are similar or identical. Additionally, hybrids have been observed in mixed colonies formed by these two species [31]. The presence of hybrids further complicates the morphometric analysis of these two species, and the introgression of the mtDNA of *M. myotis* into *M. blythii* complicates the molecular identification of the species using the *COI* fragment [31]. In order to enable the molecular identification of these two species, it is necessary to use primers for the amplification of nuclear markers, in addition to primers for the amplification of *COI* fragments [2,15]. The above example shows that, regardless of the high reliability of molecular methods, it is important to carefully approach the interpretation of the results. Nevertheless, the results of this and numerous other works indicate that metagenomic sequencing from guano samples is a powerful, non-invasive and reliable method for identifying species present at a particular location without the need to disturb the animals.

Interspecific and intraspecific distances (*d*) are calculated to determine the level of diversity at a locus, which can affect the taxonomic status of a species. The value of *d* represents the average number of base substitutions per position within sequences of the same or different species. If the value of *d* = 0, then there are no differences between different sequences, and if the value of *d* = 1, then the sequences are completely different at each nucleotide position. Bradley and Baker developed criteria for assessing taxonomic status based on genetic distances at mitochondrial loci: a distance <2% (0.02) is an indicator of intraspecific variation, distances between 2 and 11% (0.02–0.11) are an indicator of variation between closely related species, and distance values >11% (0.11) are an indicator of clear genetic separation between two species [37,38]. Genetic distances between 2 and 11% indicate the potential presence of cryptic species and require special caution when interpreting. Calculating genetic distances can be used to determine the so-called barcoding gap, which implies that there must be no overlap between the highest value of the intraspecific distance and the lowest value of the interspecific distance [15]. If a barcoding gap exists, it confirms the taxonomic status of the species at the genetic level. Relatively high interspecific distances and relatively low intraspecific distances have been recorded in bats [15,32,33], which in most cases allows for clear distinction between species at the genetic level. The results presented in this paper are consistent with the literature: all intraspecific distances are less than 2% (Table 3), except for the one recorded in *R. hipposideros*, which is 0.0229 (±0.0067) or 2.3%. The relatively high intraspecific distance in *R. hipposideros* could be explained by the fact that *R. hipposideros* is the most abundant species in B&H and accounts for a large number of sequenced samples, and therefore a higher diversity was recorded in the analyzed *COI* locus of this species compared to that of other, less abundant species.

Interspecific distances, on the other hand, are relatively high in bats, and the best illustration of this is the values of interspecific distances within the two most abundant bat genera in Europe: *Rhinolophus* and *Myotis*. The interspecific distance of the genus *Rhinolophus* in Central Europe was recorded in a range between 0.117 and 0.164, and in Russia between 0.1041 and 0.1295 [15,33]. This coincides with the results of this study, as the interspecific *d* value of the genus *Rhinolophus* in B&H ranges between 0.0427 and 0.1577 (Supplementary Table S2). Furthermore, the value of the interspecific distance of the genus *Myotis* in Central Europe ranges between 0.090 and 0.191, while in Russia it was calculated in the range between 0.0851 and 0.1363 [15,33]. A range of interspecific *d* values of 0.1182–0.2256 was calculated for *Myotis* genus in B&H, which is more in line with values recorded in Central Europe. However, it is important to note that the lengths of *COI* markers used for calculating genetic distances vary: Ruedi et al. calculated the interspecific distance between bats in Central Europe based on *COI* fragments ranging between 1545 and 700 bp [15], while Kruskop et al. calculated the interspecific distance between bats in Russia based on a *COI* fragment of 657 bp [33]. This disparity in the length of *COI* fragments means that caution is required when analyzing genetic distances reported in different studies. Despite the much shorter *COI* sequence (202 bp) used in this study, the obtained interspecific distance values are comparable to those available in the literature. In addition, it is important to note that, as the sampling area increases, the values of intraspecific distances increase as a result of population isolation by distance, while the values of interspecific distances decrease due to the sampling of a larger number of closely related species. The other two studies include a significantly larger sampling area, cover different habitats and therefore have a larger number of species recorded, which affects the values of interspecific distances [39].

If the criteria set by Bradley and Baker are applied, all species recorded in this study are genetically clearly separated from each other, i.e., have an interspecific distance >0.11. One exception is *R. mehelyi* and *R. euryale*, two closely related species whose interspecific distance is 0.0427 (±0.0138) or 4.3% which makes their taxonomic status somewhat unclear. However, it is important to emphasize that there is a barcoding gap for both species: the value of the intraspecific distance for *R. mehelyi* is 0.0178 (±0.0068) or 1.8%, while the same value for *R. euryale* is 0.005 (±0.0029) or 0.5%, and thus there is no match with the value of their interspecific distance. In addition, although *R. mehelyi* and *R. euryale* form a cluster on the phylogenetic tree (Figure 4), their branches are separated and have good bootstrap support and can be considered separate species. The low interspecific distance is potentially the result of their extremely close relationship and common ancestry, as well as overlapping habitats where gene mixing can occur as in the case of the species *M. myotis* and *M. blythii*.

It is important to emphasize that The Protected landscape Bijambare and the Dardagani quarry, two of the localities richest in biodiversity, are the sites most frequently visited by humans. The Protected landscape Bijambare has several caves, all of which are often frequented by tourists, but these visits are highly controlled, and the influence of the anthropogenic factor has been successfully limited and managed. On the other hand, Dardagani is an abandoned quarry that has already demonstrated strong anthropogenic influence, including high concentrations of heavy metals that affect the red blood cells of *Miniopterus schreibersii* [40]. In the light of the findings presented in this work, including the presence of *Rhinolophus mehelyi*, a new species for B&H, at the Dardagani quarry, the authors suggest that steps must be taken to protect these localities from further contamination and degradation due to human actions.

## 5. Conclusions

Although the study of the genetic diversity of bat species within the mitochondrial *COI* marker was not the main focus of this work, it was shown that the 202 bp *COI* fragment can be used to estimate the genetic diversity of bat populations. However, for the purposes of population genetic studies and biodiversity studies, it is necessary to use at least one nuclear marker in order to overcome the limitations resulting from the presence of similar or identical mitogenomes of different species. The results presented in this work demonstrated a high concordance of morphometric analysis with DNA barcoding, but also the superiority of the molecular method over morphometric, since it successfully identified a larger number of species. Molecular methods can successfully identify cryptic species or clarify the results of morphometric analyses if they do not provide reliable results, and by using a larger number of molecular markers, it is possible to perform population genetic analysis at a site of interest. By employing *COI* sequencing from the non-invasive guano samples, it was possible to detect a new species for B&H—*Rhinolophus mehelyi*. Then, these studies may help identify sites that are of special importance to bats by correctly estimating the biodiversity of such sites. The results indicate that protected natural areas are extremely important since they harbor the largest number of different bat species. Additionally, this study identifies a new site, an abandoned underground quarry which serves as a shelter for as many as nine different species of bats, and therefore potentially represents an area that should be considered for legal protection. In the Anthropocene, the age of constant loss of habitat largely due to human destruction, it is important to implement molecular methods in non-invasive wildlife monitoring in order to conserve the vital ecosystem functions.

## Figures and Tables

**Table 1:** Localities, species and number of individual animals of each sex.

Sampling Sites	Bat Species	Sex	No. of Individuals
Protected landscape Bijambare	*Barbastella barbastellus*	M	4
		F	1
	*Myotis* sp.	M	1
	*Rhinolophus hipposideros*	M	2
		F	6
	*Rhinolophus ferrumequinum*	F	1
Trebević	*Rhinolophus hipposideros*	M	13
		F	3
Vela	*Myotis mystacinus/alcathoe*	M	1
		F	1
Sarajevo	*Tadarida teniotis*	M	1
Dardagani quarry	*Rhinolophus hipposideros*	M	2
		F	1
	*Rhinolophus euryale*	F	1
	*Rhinolophus ferrumequinum*	M	3
		F	2
	*Myotis capaccinii*	M	2
		F	1
	*Myotis myotis*	F	7
	*Myotis myotis/blythii*	F	1
	*Miniopterus schreibersii*	M	39
		F	22
Nature monument Tajan	*Rhinolophus hipposideros*	F	1
	*Rhinolophus ferrumequinum*	F	1
Ober pit	*Rhinolophus hipposideros*	M	1
		F	1
Hrustovača cave	*Rhinolophus hipposideros*	M	7
		F	1
	*Rhinolophus ferrumequinum*	M	4
		F	3
Fajtovačka cave	*Rhinolophus hipposideros*	M	2
	*Rhinolophus ferrumequinum*	M	1
		F	2
Cave Ada	*Rhinolophus hipposideros*	M	4
		F	2
	*Rhinolophus ferrumequinum*	F	2
	*Myotis capaccinii*	F	4
	*Miniopterus schreibersii*	M	10
		F	18
Vodena cave	*Rhinolophus hipposideros*	M	3
		F	1
	*Rhinolophus ferrumequinum*	F	1
Ledenica cave	*Rhinolophus hipposideros*	M	2
Salakovac	*Rhinolophus hipposideros*	M	2
Stolac	*Rhinolophus hipposideros*	M	1

**Table 2:** Observed richness of bat species by locality as determined by morphological method and molecular (*COI*) method of species identification.

Locality Name	Samples		Observed Species Richness	
	No. of Captured Bats	No. of Samples Analyzed	Morphological Method	*COI* Analysis
Protected landscape Bijambare	15	24	4	8
Trebević	16	8	1	1
Vela	2	4	1	1
Sarajevo	1	3	1	1
Dardagani quarry	82	48	7	10
Nature monument Tajan	2	6	2	2
Ober pit	2	4	1	1
Hrustovača cave	15	18	2	3
Fajtovačka cave	5	10	2	5
Cave Ada	40	18	4	6
Vodena cave	5	11	2	4
Ledenica cave	2	10	1	2
Salakovac	2	5	1	4
Mostar	0	3	0	1
Stolac	1	4	1	6
Gradina	0	4	0	3

**Table 3:** Intraspecific genetic distances (*d*) of different bat species in B&H based on their *COI* sequences.

Family	Species	*d*	Standard Deviation
Rhinolophidae	*Rhinolophus mehelyi*	0.0178	0.0068
	*Rhinolophus hipposideros*	0.0229	0.0067
	*Rhinolophus ferrumequinum*	0.0019	0.0014
	*Rhinolophus euryale*	0.005	0.0029
Molossidae	*Tadarida teniotis*	0	0
Vespertilionidae	*Myotis mystacinus*	0.005	0.0029
	*Barbastella barbastellus*	0.005	0.0035
	*Myotis emarginatus*	0	0
	*Myotis daubentonii*	N/A	N/A
	*Myotis myotis/blythii*	0.01	0.0069
	*Vespertilio murinus*	N/A	N/A
	*Myotis capaccinii*	0.0025	0.0018
Miniopteridae	*Miniopterus schreibersii*	0	0

## Data Availability

The original data presented in the study are openly available in the BOLD Systems database at the following link: https://portal.boldsystems.org/recordset/DS-BATBIH25, accessed on 27 October 2025. The database contains all obtained *COI* sequences with supporting data (species, sampling date).

## Supplementary Materials

The following supporting information can be downloaded at: https://www.mdpi.com/article/10.3390/biology15151313/s1, Table S1: Accession numbers of 36 reference bat *COI* sequences retrieved from the BOLD Systems online database; Table S2: Interspecific genetic distances (d) of different bat species in B&H based on their *COI* sequences; Table S3: Additional information about the sampling localities; Table S4: Number of collected and analyzed samples per locality.

## Abbreviations

The following abbreviations are used in this manuscript:

B&H	Bosnia and Herzegovina
*COI*	Cytochrome C oxidase subunit I
D3	Third finger length
D5	Fifth finger length
FA	Forearm length
*nd I*	NADH dehydrogenase subunit I
